# Uncovering transgenerational phenotypic variation in *Parthenium hysterophorus* populations in Israel

**DOI:** 10.1007/s00425-026-05105-9

**Published:** 2026-08-03

**Authors:** Sahar Malka, Assaf Distelfeld, Maor Matzrafi

**Affiliations:** 1 Department of Evolutionary and Environmental Biology, University of Haifa, Haifa, Israel; 2 Department of Plant Pathology and Weed Research, Agricultural Research Organization – Volcani Institute, Newe-Ya’ar Research Center, Ramat-Yishai, Israel

**Keywords:** Agriculture, Ecology, Invasion, Parthenium weed, Phenology

## Abstract

**Main conclusion:**

Invasive *Parthenium hysterophorus* achieves rapid colonization via phenological divergence and population-specific transgenerational plasticity, balancing maternal environmental effects with rigid developmental canalization to outperform native flora across diverse habitats.

**Abstract:**

*Parthenium hysterophorus* L. is a highly aggressive invasive weed that has spread across more than 50 countries, causing severe agricultural, ecological, and public health problems. Understanding phenotypic variation among invasive populations is critical for predicting invasion dynamics and designing effective management strategies. This study investigated phenological variation and transgenerational plasticity among five *P. hysterophorus* populations in northern Israel over three years. We compared developmental traits (emergence, bolting, flowering, height, and biomass) between field-collected seeds and their progeny grown under controlled greenhouse conditions. Significant phenological differences were observed among the populations, with field populations exhibiting more uniform development. These disparities persisted through to final height and weight measurements. A detailed transgenerational analysis of two contrasting populations (Tirat Zvi and Ha'Hotrim) revealed population-specific patterns of developmental plasticity. The Tirat Zvi population, originating from an extremely arid environment and representing the earliest documented Israeli introduction, demonstrated significant transgenerational shifts in emergence timing and a 25% increase in biomass under controlled conditions, while maintaining conserved flowering timing. In contrast, the Ha'Hotrim population exhibited a limited transgenerational response, with significant changes only in bolting timing. These contrasting patterns suggest different adaptive strategies: environmental anticipation through maternal effects versus intrinsic developmental canalization. The pronounced phenological divergence among populations, despite their short geographic separation, supports the hypothesis of multiple independent introductions or rapid local adaptation, with important implications for managing this invasive species in diverse agricultural landscapes.

**Supplementary Information:**

The online version contains supplementary material available at 10.1007/s00425-026-05105-9.

## Introduction

*Parthenium hysterophorus* L. (Asteraceae), native to the Gulf of Mexico region (Rollins 1950), is a highly aggressive invasive weed that has spread to more than 50 countries (Adkins and Shabbir 2014). Its success as an invader has been attributed to its prolific seed production, competitive growth, and remarkable adaptability to diverse environments (Mahmoud et al. 2015; Cowie et al. 2022). Although typically an annual plant in its native habitat, *P. hysterophorus* may develop as a perennial in invaded regions (Mahmoud et al. 2015), demonstrating phenotypic plasticity.

The global impact of *P. hysterophorus* is severe and multifaceted. In agriculture, the weed causes substantial yield losses across various crops including maize, sorghum, sunflower, and pulses, with reductions reaching 40% in India and 81% in Ethiopia (Tamado et al. 2002; Rana et al. 2008; Bajwa et al. 2020; Dukpa et al. 2020). In Queensland, Australia, infestations spanning over 170,000 km^2^ result in annual economic losses of $16.8 million (Chippendale et al. 1994). In addition to direct competition for resources, *P. hysterophorus* serves as a secondary host for agricultural pests and pathogens, including *Phenacoccus solenopsis* and Tomato Yellow Leaf Curl Virus (TYLCV) (Kishun and Chand 1987; Arif and Ghaffar 2009; Kumar et al. 2016). Its designation on the EPPO A2 list as a quarantine species (EPPO 2022) poses significant phytosanitary risks to international trade, requiring the eradication of infested agricultural produce. In addition, *P. hysterophorus* exhibits highly allergenic properties that negatively affect both human and animal health (Towers and Rao 1992; Kohli et al. 2006). In natural ecosystems, the species displaces native flora and significantly reduces biodiversity (Sushilkumar and Ray 2010; Nguyen et al. 2017; Cowie et al. 2022). The global spread of invasive species, such as *P. hysterophorus*, has been facilitated primarily through international trade (Levine et al. 2003) and further accelerated by climate change, which enhances both their range expansion and adaptive capacity (Pyšek and Richardson 2010).

Previous studies have revealed substantial phenotypic differences among *P. hysterophorus* populations that may contribute to its invasiveness and adaptive success. In Australia, two biotypes were found to vary in allergenicity levels, which correlated with differences in competitive ability and invasiveness (Navie et al. 1996). Recently, Malka et al. (2023) investigated five Israeli populations, uncovering variations in seed characteristics, such as size and weight, as well as differential germination responses to temperature and osmotic potential. This capacity for stress adaptation is further supported by recent physiological evidence. Saravanane et al. (2023) demonstrated that *P. hysterophorus* can leverage elevated atmospheric CO2 to mitigate the inhibitory effects of soil salinity, allowing it to maintain superior growth and biomass accumulation even in degraded environments. The adaptive potential of invasive species may be further enhanced through transgenerational plasticity (TGP) the influence of parental environment on offspring phenotype which has been increasingly recognized as a mechanism facilitating invasion success (Dyer et al. 2010; Herman and Sultan 2011). Adaptive transgenerational effects appear more pronounced in invasive compared to non-invasive species (Fenesi et al. 2014), suggesting that TGP capacity may itself be under selection during invasion. Chen et al. (2024) demonstrated that high morphological transgenerational plasticity under stressful conditions accelerates the invasion of *Xanthium strumariu*, wherease stable resource allocation ensures long-term persistence. On a macroecological scale, such plasticity facilitates significant climatic niche expansion, enabling the species to colonize regions with environmental conditions distinct from its native range (Shabbir et al. 2023; Ángel-Vallejo et al. 2024). Beyond physiological tolerance, invasive success is also driven by phenological strategies, such as the exploitation of 'vacant niches' by flowering later and longer than native communities to minimize competition (Lázaro‐Lobo et al. 2025). Furthermore, these adaptive traits are not static; meta-analytical evidence suggests that key invasive traits often continue to evolve and diverge as a function of the population’s residence time in the new territory (Gruntman and Segev 2024). Therefore, determining whether these adaptive strategies stem from inherent phenotypic plasticity, transgenerational effects, or rapid evolutionary divergence is critical for understanding the differential success and potential expansion of *P. hysterophorus* in invaded habitats.

Resolving this dichotomy is particularly challenging in Israel, where the invasion history is unclear. Although *P. hysterophorus* was first detected in 1980 (Dafni and Heller 1982), the origin of *P. hysterophorus* in Israel and the number of independent introductions remain unclear. We hypothesized that distinct biotypes have independently invaded Israel and that these biotypes can be distinguished through the analysis of phenotypic traits, particularly those related to phenology and developmental patterns. Understanding the phenological and developmental characteristics of different *P. hysterophorus* populations is essential for predicting invasion dynamics, designing effective management strategies, and elucidating the mechanisms underlying successful invasions.

## Materials and methods

### Seed collection and progeny population production

Following the methodology of Malka et al. (2023), seeds were collected from five *P. hysterophorus* populations across northern Israel [Amikam (AM), Dgania Beit (DB), Ha'hotrim (HT), Newe Ya’ar (NY), and Tirat Zvi (TZ)] in the summer of 2019, based on the previous mapping by Matzrafi et al. (2021). Ripe inflorescences were harvested haphazardly from 40 mature plants at each site and bulked to define a population, in accordance with Silvertown and Charlesworth (2009). To minimize environmental maternal effects, these field-collected seeds were subsequently sown in 2 L pots and propagated in a greenhouse (35/20°C day/night) at the Newe Ya'ar Research Center. Upon reaching the reproductive stage, 30 plants from each population were isolated in pollen-proof cages to prevent cross-pollination, after which the resulting seeds were collected and bulked for progeny generation.

### Experimental timeline

Phenological evaluation was performed to conduct a comparative analysis of five Israeli populations (AM, DB, HT, NY, and TZ) and to assess their phenotypic development over 85 d after sowing. The experiment was conducted over three years. In the first year (2021), we examined plants grown from field-collected seeds (field populations). For the second year (2022), we used plants grown from progeny seeds (progeny populations), progeny seed production was described by Malka et al. (2023). Briefly, field-collected seeds were sown in a climate-controlled greenhouse, and 30 plants per population were isolated in pollen-proof cages during the bolting stage to prevent cross-pollination. At maturity, seeds were harvested and bulked by location. Both experiments, using field and progeny populations, comprised 32 plants per population. Following the first two years of the initial assessment, we chose two populations from each generation. Populations that are geographically and phenotypically different (emergence, bolting and final height) were selected for further detailed transgenerational examination in the third year (2023). The experimental setup included 38 plants per population.

### Plant development assessment

All phenological experiments were conducted in a greenhouse, maintaining an average temperature of 20/25°C (day/night). Seeds were sown in 2 L pots with planting soil (Ram 6, Tuf Merom Golan) and watered regularly with drip irrigation (2.3 L/h) adjusted for optimal watering throughout the term of the experiment. To maintain consistency, all seeds were cultivated in pots arranged in a fully randomized design. Plants were visited daily, and key stages in the plant life cycle, including emergence, bolting, and flowering, were documented. The final height and biomass were recorded at the end of the experiment for each plant.

### Statistical analysis

All statistical analyses were performed using R (version 3.3.2, i386) in the RStudio (version 1.3.1056) integrated environment (R Core Team 2020). Data visualizations based on each analysis were created using the ggplot() function of the ggplot2 package.

#### Comparison of morphological traits

Differences in averages among populations for weight and height were analyzed using the aov() function for one-way Analysis of Variance (ANOVA) To determine the significance of differences between specific pairs of populations, Tukey’s Honestly Significant Difference (HSD) post hoc tests were performed.

#### Phenological modeling

All phenological development models (emergence, bolting, and flowering) were generated using the drm() function (Onofri et al. 2018) to analyze the time-to-event Log-logistic three-parameter model. The sigmoidal Log-logistic three parameter (equation A):

$$f\left(x\right)=\frac{d}{1+\mathrm{exp}\left(b\left(\mathrm{log}\left(x\right)-\mathrm{log}\left(e\right)\right)\right)}$$

where:

$f\left(x\right)$ is the response (e.g., cumulative germination) at time x.

b is the slope at the inflection point, representing the rate of development.

d represents the upper asymptote, indicating the maximum capacity (e.g., total development percentage).

e us the inflection point (ED50), representing the time required to reach 50% of the maximum response (d).

The lower limit was fixed at 0 because negative development is biologically impossible. Parameter comparisons between populations were assessed using the compParm () function.

## Results

### Phenological response for each generation

**Fig. 1:**
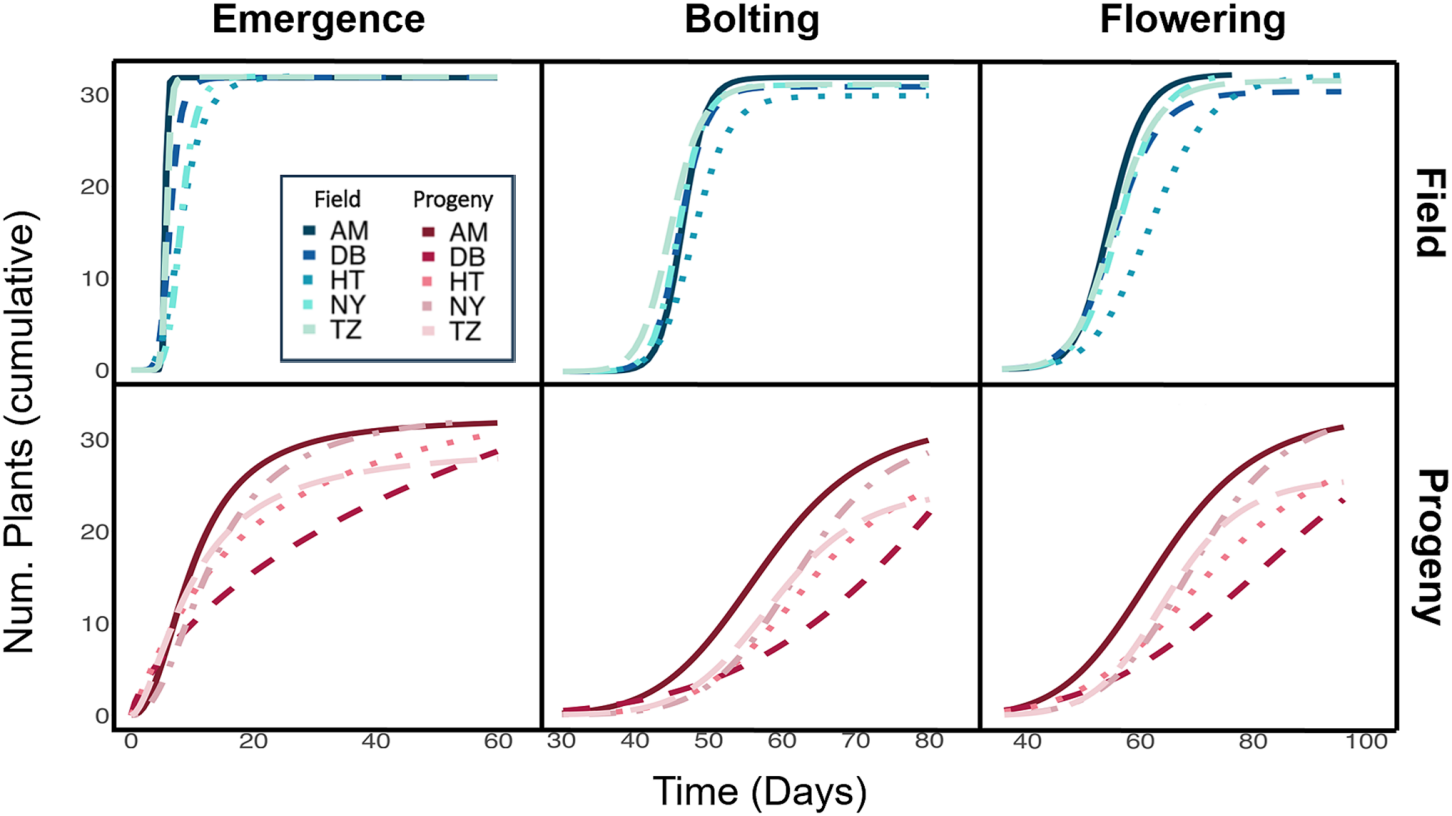
Phenological development across three main stages for five *P. hysterophorus* field (top) and progeny (bottom) populations. Model fitted using nonlinear regression with three-parameter Log-logistic curve (equation A), *n* = 32

This study was conducted over three years, following three main stages in *P. hysterophorus* life cycle in plants from field populations (2021), progeny populations (2022), and a transgenerational comparison (2023). Our results show that the field populations managed to emerge, bolt, and flower more uniformly than the progeny populations (Fig. 1).

The maximum average number of days that it took for emergence in the field populations was 13 ± 2.83 days with a total time span of 7.4 ± 3.13, while for the progeny populations, the maximum average was 48.8 ± 15.99 days with a total time span of 7.4 ± 3.13 (Table S1). Bolting for the field populations started at an average of 40 ± 0 days after sowing, and the last plants reached their bolting stage at an average of 44.8 ± 15.99 days after sowing with an average time span of 14.6 ± 3.21 days. For the progeny populations, bolting started at an average of 45.4 ± 1.34 days after sowing and reached its final at an average of 85 ± 3 days, with an average time span of 39.6 ± 3.05 days (Table S1). Flowering for the field populations started at an average of 43 ± 7.38 days after sowing and reached the final flowering plants an average of 74.6 ± 12.05 days after sowing with an average time span of 31.6 ± 12.56 days, while for the progeny populations flowering started at an average of 47.4 ± 2.3 days from sowing and reached its final an average of 90.6 ± 4.98 days, with an average time span of 43.2 ± 6.06 days (Table S1).

### Differences among populations

#### Emergence

For the field populations, significant differences between populations were observed, mainly in the time to 50% emergence (Fig. 1). Plants of TZ and AM reached maximal emergence faster with b values of −19.438 and—33.406(respectively) indicating a steeper slope while plant of DB, HT and NY populations were relatively slower with b values of −8.874, −3.750 and −6.640, respectively (Table 1). For the progeny populations, AM and NY reached maximal emergence faster with b values of −2.230 and -2.244, respectively, while DB and HT populations were relatively slower with b values of −1.181 and −1.289, respectively.

**Table 1:** Estimate of parameters for the Log-logistic three-parameter equation for the three main stages of phenological development in five *P. hysterophorus* field and progeny populations

		Population	AM			DB			HT			NY			TZ		
Generation	Stage	Parameter	Estimate	SE	*P*	Estimate	SE	*P*	Estimate	SE	*P*	Estimate	SE	*P*	Estimate	SE	*P*
Field	Emergence	b	-33.406	12.346	0.009	-8.874	1.660	< 0.0001	-3.750	0.671	< 0.0001	-6.640	1.009	< 0.0001	-19.438	7.787	< 0.0001
		d	30.891	0.645	< 0.0001	30.257	0.850	< 0.0001	35.511	4.003	< 0.0001	32.429	1.443	< 0.0001	31.284	0.696	< 0.0001
		e	5.531	0.176	< 0.0001	6.127	0.154	< 0.0001	8.787	0.733	< 0.0001	8.167	0.233	< 0.0001	5.748	0.084	< 0.0001
	Bolting	b	-29.959	2.590	< 0.0001	-26.154	2.325	< 0.0001	-21.440	2.044	< 0.0001	-25.761	1.971	< 0.0001	-20.232	1.473	< 0.0001
		d	31.848	0.484	< 0.0001	30.761	0.511	< 0.0001	29.477	0.728	< 0.0001	31.591	0.492	< 0.0001	31.991	0.515	< 0.0001
		e	46.480	0.148	< 0.0001	46.133	0.168	< 0.0001	47.945	0.239	< 0.0001	46.061	0.164	< 0.0001	45.056	0.187	< 0.0001
	Flowering	b	40.763	0.290	< 0.0001	-13.986	0.711	< 0.0001	-8.856	0.511	< 0.0001	-14.598	0.613	< 0.0001	-13.915	0.568	< 0.0001
		d	32.229	0.348	< 0.0001	30.607	0.497	< 0.0001	44.403	3.629	< 0.0001	33.174	0.490	< 0.0001	32.542	0.469	< 0.0001
		e	55.043	0.162	< 0.0001	56.017	0.241	< 0.0001	67.432	1.354	< 0.0001	57.062	0.218	< 0.0001	56.179	0.223	< 0.0001
Progeny	Emergence	b	-2.230	0.177	< 0.0001	-1.181	0.065	< 0.0001	-1.289	0.083	< 0.0001	-2.244	0.161	< 0.0001	-1.614	0.143	< 0.0001
		d	32.360	0.522	< 0.0001	36.160	1.356	< 0.0001	35.014	1.140	< 0.0001	33.174	0.609	< 0.0001	29.723	0.746	< 0.0001
		e	9.990	0.347	< 0.0001	23.450	1.811	< 0.0001	14.709	1.028	< 0.0001	13.135	0.432	< 0.0001	10.288	0.555	< 0.0001
	Bolting	b	-7.994	0.466	< 0.0001	-6.437	0.284	< 0.0001	-8.529	0.672	< 0.0001	-9.971	0.632	< 0.0001	-9.622	0.721	< 0.0001
		d	32.012	0.697	< 0.0001	35.519	1.119	< 0.0001	27.555	1.129	< 0.0001	30.841	0.783	< 0.0001	24.675	0.583	< 0.0001
		e	57.318	0.526	< 0.0001	73.780	0.867	< 0.0001	62.952	0.895	< 0.0001	62.042	0.513	< 0.0001	58.817	0.540	< 0.0001
	Flowering	b	-7.167	0.398	< 0.0001	-4.593	0.472	< 0.0001	-6.054	0.495	< 0.0001	-8.561	0.468	< 0.0001	-9.755	0.645	< 0.0001
		d	32.946	0.801	< 0.0001	40.770	8.090	< 0.0001	30.816	1.969	< 0.0001	33.512	0.875	< 0.0001	25.929	0.565	< 0.0001
		e	63.318	0.693	< 0.0001	89.768	7.615	< 0.0001	72.131	1.952	< 0.0001	69.738	0.650	< 0.0001	64.796	0.546	< 0.0001

Model fitted using nonlinear regression with three-parameter Log-logistic curve (equation A), *n* = 32; SE, standard error

When comparing the significant differences in the *e* parameter (time to 50% emergence) for the field populations, NY (*e* = 8.167) demonstrated a significant difference with AM (*e* = 5.531) and DB (*e* = 6.127), which reached 50% emergence faster, and HT (*e* = 8.787), which reached 50% emergence slower (*P* < 0.008, *P* < 0.0001, *P* < 0.0001, respectively, Table S2).

For the progeny populations, HT (*e* = 14.709) showed a significant difference in the *e* parameter when compared with the AM (*e* = 9.990) and TZ (*e* = 10.288) populations (*P* < 0.005 and *P* < 0.004, respectively; Table S2).

#### Bolting

Bolting behavior in field populations was relatively similar. Plant of AM, DB and NY populations showed steeper slopes (b = −29.959, −26.154, −25.761 respectively, Table 1) meaning faster bolting rate at the point of *e* parameter, although time to 50% bolting was relatively similar for all populations with *e* values of 46.480, 46.133, 46.061 and 45.056 days for AM, DB, NY and TZ, respectively. While HT plants showed the latest timing for 50% bolting with a value of 47.945 days (Table 1), all field population comparisons other than DB/TZ and DB/AM were significant (*P* < 0.0001) for the time to 50% bolting analysis (Table S2). This indicates that while the overall timeframe for bolting was narrow across all field populations, there were statistically significant differences in the precise timing of 50% bolting between specific populations, with the HT population representing a statistically unique late-bolting phenotype.

For the progeny populations, the time to 50% bolting was the shortest for AM and TZ, with 57.318 and 58.817 days, respectively. Although DB lagged as compared to all other populations, with a value of 73.780 days (Table 1). Plants of the HT progeny population (*e* = 62.952) showed a significant difference in 50% bolting when compared with the AM and TZ populations (*P* < 0.0001, Table S2).

#### Flowering

Plants of the field population AM reached maximal flowering relatively faster with b value of −17.667 indicating a steeper slope, while plants of DB, NY and TZ showed b values of −13.986, −14.589 and −13.915, respectively, and HT plants had a prolonged flowering time with a slope of −8.856 (Table 1). This was also reflected by the *e* values, which showed that plants from AM, DB, NY, and TZ took 55.043, 56.017, 57.062, and 56.179 days, respectively, to reach 50% flowering, while HT plants reached 50% flowering after 67.432 days (Table 1). For field populations, when comparing the differences in 50% time to flowering, the NY and TZ populations showed significant differences (*P* < 0.0001, Table S2) when compared with all other populations, including each other. This indicates that phenological divergence in flowering time is highly significant among these populations, ranging from the early flowering AM population to the distinctly late-flowering HT population.

For the progeny populations, TZ plants showed the steepest slope (*b* = −9.755), whereas the time to 50% flowering was relatively high (64.796 days). AM plants showed a more moderate slope (*b* = −7.167) and a shorter time to 50% flowering of 63.318 days (Table 1). Plants of the DB population had a gradual slope with *a b* value of −4.593 and the longest timing for 50% flowering of 89.768 days (Table 1). When comparing the time to 50% flowering between plant populations, there were significant differences for the HT population in comparison with the AM and TZ populations (*P* < 0.0001 and *P* < 0.002, respectively, Table S2), while TZ plants also showed significant differences in comparison with the DB and NY populations (*P* < 0.002 and *P* < 0.0001, respectively, Table S2). This means that the progeny populations exhibit significant transgressive segregation or divergence in flowering phenology, with specific lineages, such as the DB population, displaying a delayed reproductive onset as compared to other populations.

#### Height and biomass

**Fig. 2:**
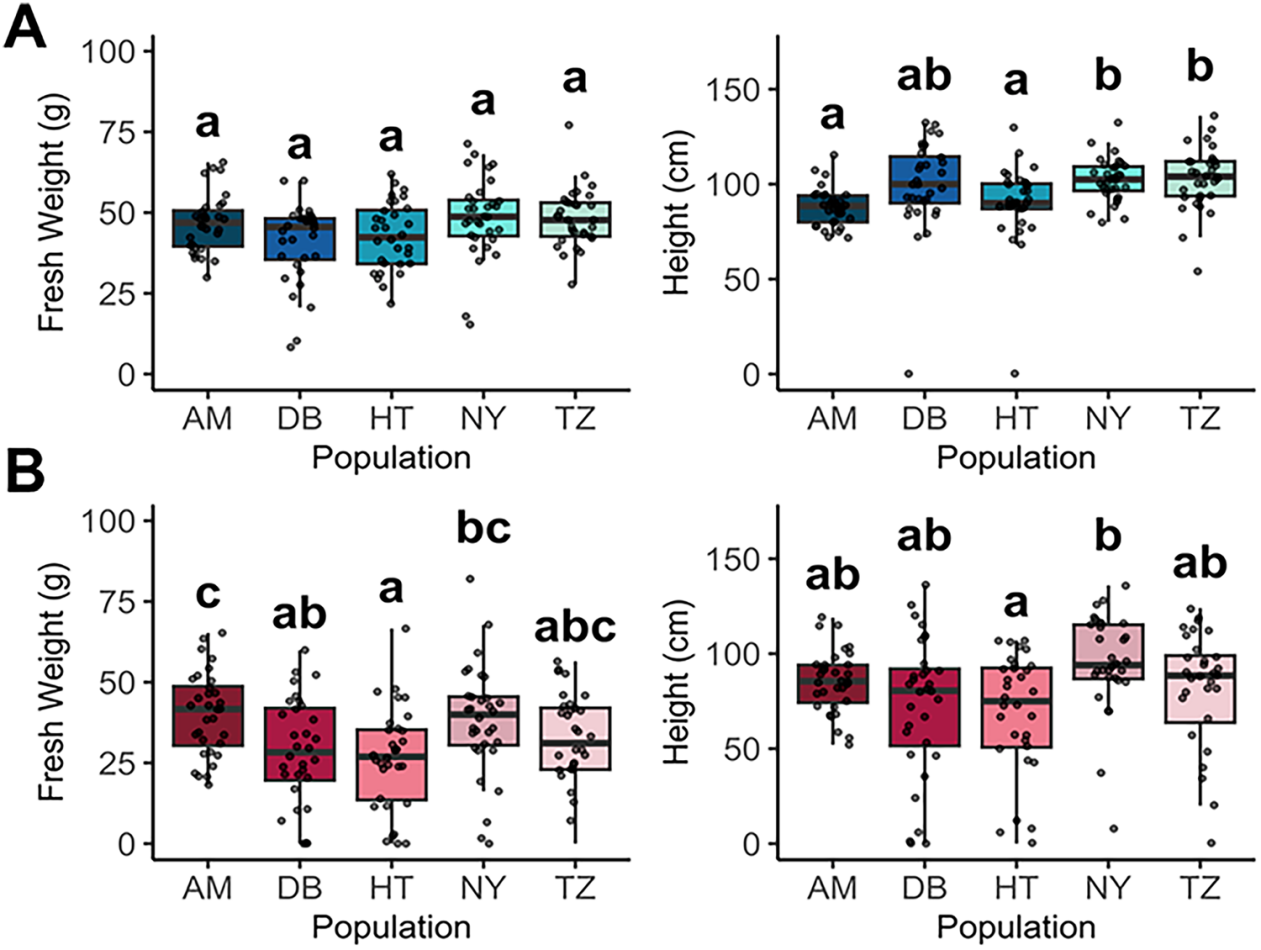
Final fresh weight and height of five *P. hysterophorus* field (**A**) and progeny (**B**) populations. Statistically significant by Tukey HSD (*P* < 0.05, *n* = 32)

For fresh weight, no significant differences between field populations were observed (Fig. 2A), whereas plant height showed two significant groups. The final mean height of plants from the AM (87.969 ± 1.777, Table S3) and HT (90.063 ± 3.689, Table S3) populations were significantly different from plants of NY (102.156 ± 1.995, Table S3) and TZ (102.906 ± 2.888, Table S3), while DB (99.469 ± 4.324, Table S3) showed an intermediate response (Fig. 2A).

Higher diversity was recorded in the progeny populations (Fig. 2B). The final mean weight of plants in the HT population (26.226 ± 2.808, Table S3) was the lowest and significantly different from that of the AM (41.126 ± 2.289, Table S3) and NY (38.067 ± 3.077) populations. In contrast, the TZ average final weight (30.935 ± 2.809, Table S3) showed no significant difference from any other population. Progeny plants final average height showed one main significant difference between HT (67.563 ± 5.864, Table S3) and NY (92.844 ± 5.427, Table S3), while all other populations showed no significant difference.

## Transgenerational variation

The third part of this study examined the differences between the two plant populations, TZ and HT, at the developmental stages of both field and progeny seed populations, exploring the transgenerational impact.

### Emergence

**Fig. 3:**
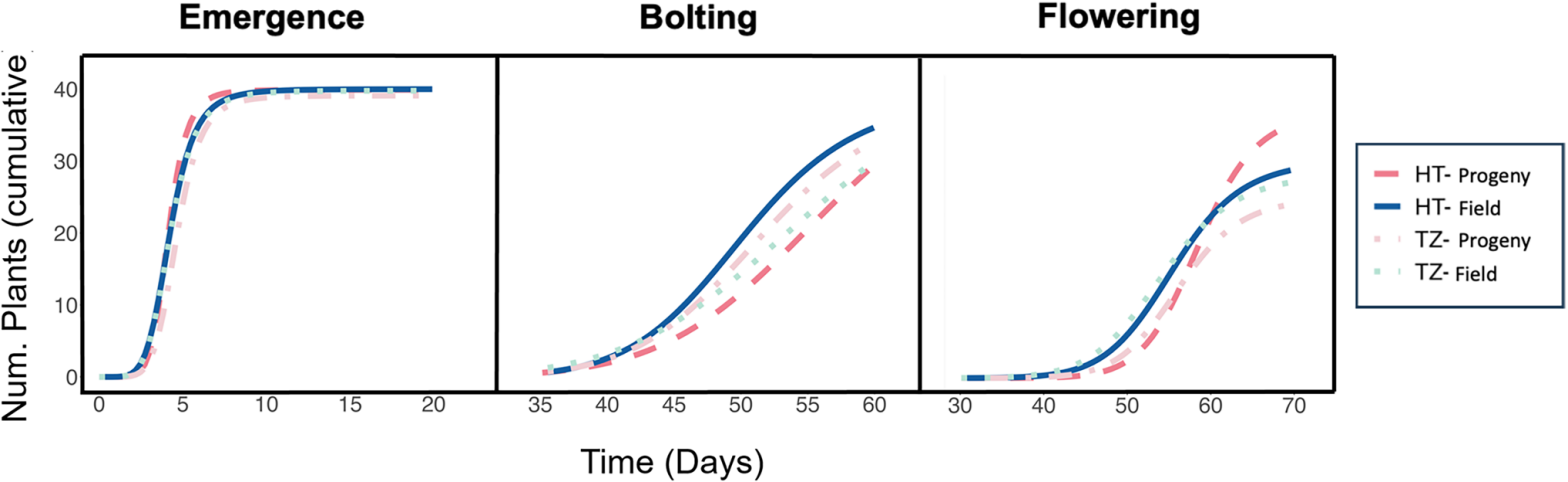
Phenological development in three main stages of the two selected *P. hysterophorus* field and progeny populations. Model fit using nonlinear regression with Log-logistic three-parameter curve (equation A), *n* = 40

Figure 3 shows a relatively uniform emergence between populations; however, several differences were observed. Emergence of all populations from both generations started 4 days after sowing, and seedling emergence in both the HT field and progeny populations continued until 9 days after sowing. Seedlings from the TZ field population showed maximum emergence at day 13, while seedlings from the TZ progeny population took 26 days from sowing (Table S4). Plants from the HT progeny population showed a steeper slope (*b* = −7.409, Table 2) than those from the HT field population (*b* = −5.667, Table 2), although their time to 50% emergence was similar (*e* = 4.225, 4.131, field and progeny, respectively) and showed no significant difference (*P* < 0.290, Table S5). Plants from the TZ progeny population showed a steeper slope (*b* = −6.426, Table 2) than plants from the TZ field population (*b* = −5.670, Table 2), while their time to 50% emergence was similar in value (*e* = 4.251, 4.634, field and progeny, respectively) and significantly different (*P* < 0.0001, Table S5). This indicates that while the speed of emergence increased in the progeny generation for both TZ and HT populations, only the TZ population showed a statistically significant shift in the actual timing of 50% emergence. While the slope (*b*) of emergence increased in the progeny generation for both TZ and HT populations, only the TZ population showed a statistically significant transgenerational shift in the actual timing of 50% emergence.

**Table 2:** Estimate of parameters for the Log-logistic three-parameter equation for the three main stages of phenological development in the two selected *P. hysterophorus* parental and progeny populations.

			Emergence			Bolting			Flowering		
Population	Generation	Parameter	Estimate	SE	*P*	Estimate	SE	*P*	Estimate	SE	*P*
HT	Progeny	b	-7.409142	0.639371	< 0.0001	-9.06632	0.7867	< 0.0001	-16.9446	3.9596	< 0.0001
		d	39.898761	0.273012	< 0.0001	46.19024	2.31401	< 0.0001	36.9958	3.7631	< 0.0001
		e	4.130949	0.0575	< 0.0001	56.32813	0.82624	< 0.0001	58.5539	1.1322	< 0.0001
	Field	b	-5.666663	0.403259	< 0.0001	-11.60666	0.95292	< 0.0001	-13.4142	2.716	< 0.0001
		d	39.997263	0.284831	< 0.0001	39.05302	1.09161	< 0.0001	30.2507	2.5613	< 0.0001
		e	4.225323	0.067536	< 0.0001	50.14122	0.4421	< 0.0001	55.4625	1.231	< 0.0001
TZ	Progeny	b	-6.426139	0.503609	< 0.0001	-10.57207	0.87955	< 0.0001	-15.1093	4.1062	< 0.0001
		d	39.100411	0.282943	< 0.0001	39.41892	1.39146	< 0.0001	25.0864	2.6562	< 0.0001
		e	4.63356	0.067217	< 0.0001	51.5697	0.56399	< 0.0001	56.1855	1.431	< 0.0001
	Field	b	-5.670414	0.42141	< 0.0001	-8.08036	0.7584	< 0.0001	-12.5011	2.8542	< 0.0001
		d	39.781677	0.2864	< 0.0001	44.15344	2.80938	< 0.0001	28.3907	2.504	< 0.0001
		e	4.25056	0.067871	< 0.0001	54.68048	1.11861	< 0.0001	54.1875	1.3286	< 0.0001

Model fitted using nonlinear regression with three-parameter Log-logistic curve (equation A), *n* = 40; SE, standard error

### Bolting

In the bolting developmental stage, there were visible differences (Fig. 3) between populations, with the HT field population reaching maximal bolting faster, with a *b* value of −11.607, in comparison to plants from the HT progeny population, which showed a *b* value of −9.066 (*P* < 0.0001, Table S5). Moreover, plants of the HT field population reached 50% bolting at 50.141 days, while plants of the HT progeny population took 56.328 days after emergence (Table 2). Plants from the TZ populations showed different slope values, with a steeper slope for TZ progeny with a *b* value of -10.572 and a less steep slope for the TZ field population with a *b* value of -8.080. However, there was a significant difference (*P* < 0.014, Table S5) in the time to 50% bolting. This shows that transgenerational effects manifested differently across populations: HT progeny exhibited a significant developmental lag in bolting compared to their field population, and TZ progeny exhibited a significantly steeper bolting rate, suggesting increased developmental synchrony compared to their field generation.

### Flowering

Analysis of the flowering developmental stage (Fig. 3) revealed distinct patterns. Although the HT progeny population exhibited a slower initial onset, it ultimately reached the highest total number of flowering plants. This trend is reflected in the *b* values, as HT progeny showed the steepest slope with a *b* value of -16.945 (Table 2). Although there were different flowering patterns, there were no significant differences in the time to 50% flowering (*e*) between the field and progeny generations within each population (Table S5; HT: *P* < 0.067, TZ: *P* < 0.309). This indicates that, although visible differences existed in both the time to 50% flowering and the final percentage of flowered plants, the primary timing parameter (*e*) remained statistically conserved across generations. This conservation is further supported by the maximum time to flowering, which remained consistent at 71 days for the TZ Field, TZ progeny, and HT field populations and 67 days for HT progeny, demonstrating that the total time span remained relatively conserved across populations (Table S4).

### Final weight and height

**Fig. 4:**
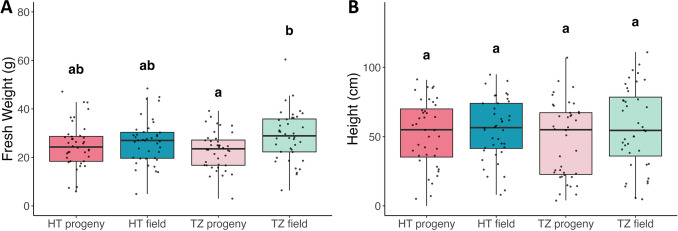
Final fresh weight (A) and final height (B) of the two selected *P. hysterophorus* parental and progeny populations. Statistically significant by Tukey HSD (*P* < 0.05, *n* = 40)

In terms of final weight (Fig. 4), there was no difference between the HT field (24.500 ± 1.456) and progeny (26.553 ± 1.593) populations, whereas there was a significant difference between the TZ field (23.013 ± 1.625) and progeny (28.848 ± 1.219) populations (Table S6). The final height showed no significant differences between either population or generation (Fig. 4).

## Discussion

The significant differences in developmental timing across the five Israeli populations support the hypothesis of distinct biotypes within Israel. Similar phenotypic differentiation has been documented in other invaded ranges. Kaur et al. (2023) identified distinct winter and summer biotypes in Indian *P. hysterophorus* populations, attributing the observed intraspecific variation to plasticity or a combination of genetic and environmental (G × E) factors. In Australia, two biotypes varying in allergenicity and competitive ability have been characterized (Navie et al. 1996), with genotypic studies confirming their origin from separate introduction events (Jabeen et al. 2015). The magnitude of phenological divergence observed among these Israeli populations, despite their relatively short geographic separation, strongly supports a similar multiple introduction scenario. Typically, a single introduction results in a genetic bottleneck that limits the standing genetic variation available for a rapid divergence. However, the maintenance of distinct, heritable biotypes (e.g., the specific biomass allocation of HT versus the unique phenology of TZ) argues against a single genetically uniform founding population. Furthermore, Israel's position as a trade hub with multiple potential entry vectors (Mediterranean Sea ports vs. Jordan Valley land borders) provides a geographic opportunity for distinct propagule pressures, potentially seeding genetically diverse populations pre-adapted to different local conditions.

Environmental data from the collection sites (Table S6) provide important context for interpreting these phenological differences and may suggest rapid adaptation to these habitats. The TZ and DB field populations originated from the most extreme sites characterized by the highest average temperatures (22.15°C and 22.28°C, respectively), lowest annual rainfall (270.6 mm and 388.7 mm), lowest relative humidity (65.7% and 66.6%), and arid serozem soils. These field populations exhibited distinct developmental patterns compared to those from cooler, more humid sites. Notably, the TZ field population showed a unique flowering pattern, whereas the DB field population displayed characteristics that distinguished it from other field populations. Among the progeny populations, the DB progeny exhibited delayed bolting and flowering compared to all other progeny populations. The capacity of P*. hysterophorus* to tolerate environmental extremes is supported by recent physiological findings. Saravanane et al. (2023) demonstrated that this species can leverage elevated atmospheric CO₂ levels to mitigate salinity stress, maintaining superior growth even in degraded environments. Such physiological plasticity may underpin the success of populations in harsh saline serozem habitats.

In contrast, the AM, HT, and NY field populations originating from sites with more moderate temperatures (20.1–20.4°C), higher rainfall (584–634 mm), higher relative humidity (72–73%), and nutrient-rich rendzina and grumusol soils (Table S6) displayed different phenological strategies among field populations. The NY field population showed the earliest emergence and flowering among the field populations, whereas the AM progeny population reached maximal bolting and flowering the fastest among all progeny populations. The correlation between the source environment and phenological behavior suggests that populations may have adapted to their local climatic conditions, potentially employing different developmental strategies. This pattern is consistent with the broader evidence that invasive plant populations rapidly evolve along climatic gradients. Colautti and Barrett (2013) demonstrated that *Lythrum salicaria* evolved distinct phenological clines within decades of introduction, with populations at invasion fronts showing increased fitness through locally adapted flowering times. Similarly, trait differentiation along temperature and precipitation gradients has been documented in the invasive *Sonchus oleraceus*, where introduced populations showed adaptive shifts in phenology and growth traits correlated with the local climate (Ollivier et al. 2020). However, the soil type variation, ranging from serozems in the drier sites to rendzinas and grumusols in wetter sites, adds complexity, as edaphic factors may also drive selection for different root development and water acquisition strategies that influence above-ground phenology (Annapurna and Singh 2003).

The lack of significant differences in biomass among field populations contrasts sharply with the greater variation observed in progeny populations. The emergence of biomass differentiation in progeny populations under controlled common garden conditions likely reveals genetic variation that is environmentally constrained in field settings. Similar patterns have been observed in other invasive ranges of *P. hysterophorus* in other countries. For example, Kaur et al. (2023) found that morpho-functional trait differences in *P. hysterophorus* biotypes became apparent only in mature flowering plants under controlled conditions, whereas metabolic differences were manifested during early growth.

The phenological variation observed among populations may contribute to the invasion success across Israel's diverse agricultural landscapes. Phenological strategies have been increasingly recognized as key determinants of the establishment and spread of invasive plants (Wolkovich and Cleland 2014; Molofsky et al. 2022). Molofsky et al. (2022) proposed that successful invaders possess phonological characteristics that are "optimally differentiated" from native flora distinct enough to avoid competitive exclusion but similar enough to pass environmental filtering. Extending this framework to intraspecific variation, different *P. hysterophorus* biotypes may be pre-adapted to different cropping systems, sowing dates, or microclimates within Israel. The distinct emergence timing of the NY field populations (significantly different from the AM, DB, and HT field populations) and the unique flowering pattern of the TZ field population could enable these biotypes to exploit temporal niches unavailable to other populations. This is consistent with the "vacant niche" hypothesis, wherein invasive plants succeed by flowering later or longer than native communities to minimize competition (Lázaro‐Lobo et al. 2025). Temporal niche separation is further facilitated by the reproductive biology of these species; *P. hysterophorus* is known to be anemophilous (wind-pollinated) and capable of autogamy, allowing it to reproduce effectively even in the absence of specific insect pollinators (Usharani and Solomon Raju 2018).

Transgenerational analysis focusing on HT and TZ populations provides particularly compelling evidence for population-level differences in developmental plasticity. These two populations were selected for detailed field-progeny comparisons because of their contrasting environments and potentially different invasion histories, with TZ representing the founding population in Israel (first detected in 1980) and HT representing a more recently documented location of introduction.

The HT population exhibited significant differences between the field and progeny generations, specifically in bolting timing, where field plants reached 50% bolting significantly earlier than progeny plants. This 6-day delay in progeny bolting timing suggests that the controlled common garden environment revealed a slower intrinsic developmental rate when maternal field effects were removed. Importantly, the HT field and progeny populations maintained consistent emergence and flowering timing, indicating developmental stability in these critical growth stages, despite environmental differences. The biomass of HT plants also remained statistically similar between the field and progeny generations, suggesting consistent resource allocation patterns. This pattern of selective plasticity, in which bolting timing responds to environmental conditions while other traits remain canalized, may reflect either a recently introduced genotype that has not yet evolved extensive condition-dependent plasticity or a phenotype specifically adapted to its coastal habitat that benefits from developmental stability in emergence and reproduction while maintaining flexibility in vegetative development.

In contrast, the TZ population demonstrated a markedly different transgenerational response. The TZ field and progeny populations differed significantly in emergence timing, with progeny plants emerging later than field-collected seeds, a delay that suggests substantial maternal environmental conditioning. Additionally, TZ progeny plants achieved significantly higher final biomass than field plants, representing a 25% increase in biomass production under controlled conditions. However, the TZ field and progeny populations maintained remarkably consistent flowering timing, indicating strong canalization of reproductive timing despite vegetative plasticity. This pattern suggests an evolved bet-hedging strategy (Cohen 1966), in which TZ populations maintain flexibility in vegetative traits (emergence timing and growth potential), sacrificing short-term success while maximizing long-term survival by preserving reliable reproductive timing to ensure seasonal fitness.

The contrasting transgenerational patterns between the HT and TZ populations are particularly striking when considering their environmental origins and establishment histories. TZ's greater developmental plasticity in the vegetative stages, combined with its significant biomass increase under benign conditions, suggests that this population has evolved adaptive responses to environmental unpredictability over more than 40 years of establishment in one of Israel's most extreme habitats. The ability of TZ progeny to achieve higher biomass when released from maternal stress signals indicates considerable growth potential that may be environmentally constrained under harsh field conditions. This pattern aligns with adaptive transgenerational plasticity theory, where offspring performance is enhanced when parental stress signals are removed (Dyer et al. 2010; Herman and Sultan 2011).

Conversely, HT's more limited transgenerational response, significant only in bolting timing while maintaining consistency in emergence, flowering, and biomass, suggests either a more recently established population with less evolved plasticity or a population adapted to its specific coastal environment that benefits from developmental canalization. The delayed bolting in HT progeny compared to field plants contrasts sharply with TZ's pattern and may reflect different adaptive strategies. While TZ appears to employ environmental anticipation through maternal effects, HT may rely on intrinsic developmental programs that are less dependent on environmental cues. Our findings align with recent evidence that transgenerational plasticity (TGP) drives the success of invasive species. As demonstrated in *Xanthium strumarium*, high morphological plasticity under stress accelerates expansion, whereas stable resource allocation ensures persistence (Chen et al. 2024). Similar adaptive maternal effects, such as drought-primed offspring tolerance in *Helianthus annuus* (Vancostenoble et al. 2022) and salinity-triggered germination shifts in *Medicago truncatula* (Vu et al. 2015), parallel our TZ and DB results, suggesting that environmentally cued phenotypic modulation is a convergent invasion strategy.

## Conclusions

This study revealed substantial phenological variation among *P. hysterophorus* populations in Israel, both between geographically distinct populations and across generations. The observed differences in developmental timing, synchrony, and biomass allocation provide important insights into the invasion dynamics of this aggressive species. Our findings of pronounced generational effects alongside population-specific phenological patterns suggest a complex interplay between genetic differentiation and environmental conditioning that shapes the success of **P. hysterophorus** in Israeli agricultural landscapes. The correlation between source environment characteristics (temperature, rainfall, humidity, and soil type) and phenological behavior, combined with the maintenance of population differences under common garden conditions, supports the hypothesis of locally adapted biotypes. Whether this differentiation arose through multiple independent introductions or rapid post-introduction evolution remains an open question with important implications for the management of this species.

## Supplementary Information

Below is the link to the electronic supplementary material.

Supplementary file1 (DOCX 30 kb)

## Data Availability

Data will be made available upon reasonable request from Dr. Matzrafi.

## Abbreviations

AM: Amicon population
DB: Dgania Beit population
HT: Ha'hotrim population
NY: Newe Ya’ar population
TZ: Tirat Zvi population
