# Supplementary material for: Uncovering transgenerational phenotypic variation in Parthenium hysterophorus populations in Israel
Source: Planta. 2026 Aug 3;264(3):83. doi: 10.1007/s00425-026-05105-9 (PMC13433396; doi:10.1007/s00425-026-05105-9)
Supplement: Supplementary file 1 [file 425_2026_5105_MOESM1_ESM.docx]

**Supplementary**

**Table S1.** Phenological development timing across three main stages in field and progeny populations of *P. hysterophorus*. Values represent the minimum and maximum day from sowing at which plants reached emergence, bolting, or flowering, and the total time span between the first and last individual reaching each stage within a population. *n* indicates the number of plants that reached the respective stage; generation-level values are shown as mean ± SD across populations.

|  |  | Emergence | | | | Bolting | | | | Flowering | | | |
| --- | --- | --- | --- | --- | --- | --- | --- | --- | --- | --- | --- | --- | --- |
| Generation | Population | Minimum (days) | Maximum (days) | total time span (days) | n. plants | Minimum (days) | Maximum (days) | total time span (days) | n. plants | Minimum (days) | Maximum (days) | total time span (days) | n. plants |
| Field | AM | 4 | 14 | 10 | 32 | 40 | 57 | 17 | 32 | 47 | 69 | 22 | 32 |
|  | DB | 6 | 15 | 9 | 32 | 40 | 55 | 15 | 31 |  | 71 | 41 | 31 |
|  | HT | 6 | 14 | 8 | 32 | 40 | 58 | 18 | 30 | 47 | 96 | 49 | 30 |
|  | NY | 6 | 14 | 8 | 32 | 40 | 53 | 13 | 31 | 47 | 70 | 23 | 32 |
|  | TZ | 6 | 8 | 2 | 32 | 40 | 50 | 10 | 31 | 44 | 67 | 23 | 31 |
|  | mean ± SD | 5.6± 0.89 | 13±2.83 | 7.4±3.13 | 32±0 | 40±0 | 54.6±3.21 | 14.6±3.21 | 31±0.71 | 43±7.38 | 74.6±12.05 | 31.6±12.56 | 31.2±0.84 |
| Progeny | AM | 4 | 34 | 30 | 32 | 44 | 82 | 38 | 31 | 46 | 86 | 40 | 31 |
|  | DB | 4 | 63 | 59 | 32 | 44 | 88 | 44 | 28 | 49 | 88 | 39 | 22 |
|  | HT | 4 | 38 | 34 | 32 | 46 | 85 | 39 | 27 | 44 | 96 | 52 | 27 |
|  | NY | 4 | 40 | 36 | 32 | 46 | 82 | 36 | 29 | 49 | 87 | 38 | 30 |
|  | TZ | 4 | 69 | 65 | 32 | 47 | 88 | 41 | 27 | 49 | 96 | 47 | 25 |
|  | mean ± SD | 4±0 | 48.8±15.99 | 44.8±15.99 | 30.6±1.34 | 45.4±1.34 | 85±3 | 39.6±3.05 | 28.4±1.67 | 47.4±2.3 | 90.6±4.98 | 43.2±6.06 | 27±3.67 |

SD = standard deviation.

**Table S2.** Statistical differences in the time to 50% of maximal occurrence for three main developmental stages in *P. hysterophorus* field and progeny populations.

| Population | Field | | | Progeny | | |
| --- | --- | --- | --- | --- | --- | --- |
|  | Emergence | bolting | Flowering | Emergence | bolting | Flowering |
|  | *P* | *P* | *P* | *P* | *P* | *P* |
| AM/DB | 0.590 | 0.016 | 0.605 | 0.278 | 0.009 | 0.001 |
| AM/HT | 0.184 | <0.0001 | 0.097 | 0.005 | <0.0001 | <0.0001 |
| AM/NY | 0.008 | <0.0001 | <0.0001 | <0.0001 | <0.0001 | <0.0001 |
| AM/ TZ | 0.681 | <0.0001 | <0.0001 | 0.857 | 0.039 | 0.104 |
| DB/HT | <0.0001 | <0.0001 | 0.231 | 0.312 | 0.020 | 0.024 |
| DB/NY | <0.0001 | <0.0001 | <0.0001 | 0.290 | 0.017 | 0.024 |
| DB/TZ | 0.931 | 0.223 | <0.0001 | 0.278 | 0.011 | 0.002 |
| HT/NT | 0.000 | <0.0001 | <0.0001 | 0.086 | 0.331 | 0.915 |
| HT/TZ | 0.266 | <0.0001 | <0.0001 | 0.004 | <0.0001 | 0.002 |
| NT/TZ | 0.016 | <0.0001 | <0.0001 | <0.0001 | <0.0001 | <0.0001 |

**Table S3.** Descriptive statistics underlying boxplot visualizations (Figure 2) of fresh weight (g) and height (cm) across five *P. hysterophorus* populations (AM, DB, HT, NY, TZ) and two generations (Field and Progeny).

| Generation | Trait | Population | Mean | SE |
| --- | --- | --- | --- | --- |
| Field | Fresh Weight | AM | 46.619 | 1.578 |
|  |  | DB | 40.794 | 2.186 |
|  |  | HT | 41.563 | 2.255 |
|  |  | NY | 48.594 | 2.186 |
|  |  | TZ | 46.847 | 2.183 |
|  | Height | AM | 87.969 | 1.777 |
|  |  | DB | 99.469 | 4.324 |
|  |  | HT | 90.063 | 3.689 |
|  |  | NY | 102.156 | 1.995 |
|  |  | TZ | 102.906 | 2.888 |
| Progeny | Fresh Weight | AM | 40.126 | 2.289 |
|  |  | DB | 28.275 | 2.982 |
|  |  | HT | 26.226 | 2.808 |
|  |  | NY | 38.067 | 3.077 |
|  |  | TZ | 30.935 | 2.809 |
|  | Height | AM | 85.625 | 2.918 |
|  |  | DB | 72.5 | 6.591 |
|  |  | HT | 67.563 | 5.864 |
|  |  | NY | 92.844 | 5.427 |
|  |  | TZ | 78.25 | 6.296 |

SE – standard error.

**Table S4.** Phenological development timing across three main stages in field and progeny populations of *P. hysterophorus*. Values represent the minimum and maximum day from sowing at which plants reached emergence, bolting, or flowering, and the total time span between the first and last individual reaching each stage within a population. *n* indicates the number of plants that reached the respective stage.

|  |  | Emergence | | | | Bolting | | | | Flowering | | | | |
| --- | --- | --- | --- | --- | --- | --- | --- | --- | --- | --- | --- | --- | --- | --- |
| Population | Generation | Minimum (days) | Maximum (days) | total time span (days) | n. plants | Minimum (days) | Maximum (days) | total time span (days) | n. plants | Minimum (days) | Maximum (days) | total time span (days) | n. plants |  |
| TZ | Field | 4 | 13 | 9 | 40 | 40 | 65 | 25 | 36 | 42 | 71 | 29 | 34 |  |
|  | Progeny | 4 | 26 | 22 | 40 | 39 | 69 | 30 | 37 | 43 | 71 | 28 | 32 |  |
| HT | Field | 4 | 9 | 5 | 40 | 40 | 61 | 21 | 37 | 45 | 71 | 26 | 37 |  |
|  | Progeny | 4 | 9 | 5 | 40 | 39 | 65 | 26 | 38 | 42 | 67 | 25 | 35 |  |

Table S5. Estimate of *e* parameter comparison of the log logistic three-parameter equation (equation A) phenological development in three main stages of two *P. hysterophorus* field and progeny populations. *e*- infliction point or where 50% of maximal development has accrued.

| Population | Emergence | Bolting | Flowering |
| --- | --- | --- | --- |
|  | *P* | *P* | *P* |
| HT progeny-HT field | 0.290 | <0.0001 | 0.067 |
| HT progeny-TZ progeny | <0.0001 | <0.0001 | 0.197 |
| HT progeny-TZ field | 0.182 | 0.239 | 0.014 |
| HT field-TZ progeny | <0.0001 | 0.049 | 0.702 |
| HT field-TZ field | 0.793 | <0.0001 | 0.483 |
| TZ progeny-TZ field | <0.0001 | 0.014 | 0.309 |

Table S6. Descriptive statistics underlying boxplot visualizations (Figure 4) of fresh weight (g) and height (cm) across populations (HT and TZ) and generations (Field and Progeny).

| Trait | Population | Generation | Mean | SE |
| --- | --- | --- | --- | --- |
| Fresh Weight | HT | Field | 24.500 | 1.4558 |
|  |  | Progeny | 26.553 | 1.5931 |
|  | TZ | Field | 23.013 | 1.6254 |
|  |  | Progeny | 28.848 | 1.2188 |
| Height | HT | Field | 51.450 | 3.4596 |
|  |  | Progeny | 55.100 | 3.9285 |
|  | TZ | Field | 47.825 | 4.6164 |
|  |  | Progeny | 56.650 | 4.3095 |

SE – standard error.

Table S7. Environmental conditions data for all five *P. hysterophorus* seed collection sites, at the year of collection (2019). Soil types were gathered from a soil group map of the Israeli Agricultural ministry; Temperature, relative humidity and rainfall were collected form the Israeli metrological services archives and open data source. ± stands for the SE.

| Population | Location in Israel | Coordination | Soil Type | Temperature (annual average) | | | Relative humidity | Rainfall (annual average) |
| --- | --- | --- | --- | --- | --- | --- | --- | --- |
|  |  |  |  | Day | Night | Average |  |  |
| AM | Amikam | 32.540416, 35.009101 | Brown and pale rendzina | 22.240±0.17 | 17.752±0.14 | 20.096±0.12 | 72.526±0.38 | 634.3 |
| DB | Dgania Beit | 32.702997, 35.581207 | Lime serozems, hydromorphic Calcareous grumusols | 25.158±0.21 | 19.400±0.18 | 22.278±0.15 | 66.591±0.42 | 388.7 |
| HT | Ha'hotrim | 32.749233, 34.955718 | alluvial arid grumusol and sand dunes | 22.795±0.16 | 18.110±0.14 | 20.449±0.15 | 73.358±0.34 | 604.3 |
| NY | Newe Ya'ar | 32.721746, 35.192867 | Brown grumusol and brown rendzina | 23.260±0.19 | 17.558±0.15 | 20.140±0.13 | 72.214±0.39 | 584 |
| TZ | Tirat Zvi | 32.426831, 35.523965 | Serozems Calcareous | 25.097±0.22 | 19.211±0.18 | 22.152±0.15 | 65.744±0.44 | 270.6 |
